# Engineering Sexless Seeds as a Path to High-Yield Crops

**DOI:** 10.1371/journal.pbio.1000118

**Published:** 2009-06-09

**Authors:** Robin Meadows

**Affiliations:** Freelance Science Writer, Fairfield, California, United States of America

Global food security is hampered by the fact that today's high-yield grains are hybrids, with seeds that fail to retain all of the desired traits, necessitating ongoing rounds of breeding and selection. In contrast, seeds produced asexually are, effectively, clones of the mother plant and so retain the desired traits, greatly streamlining their propagation. Asexual seed production (apomixis) occurs naturally in some 400 species of flowering plants, most commonly in the grass, sunflower, rose, and citrus families. However, the phenomenon is rare in commercially important crops apart from apples, mangoes, and citrus, and conventional breeding has yet to extend apomixis to other crop types.

Genetic engineering may be a more promising approach to developing apomictic crops.

The first step is stopping meiosis, the type of cell division that produces male and female gametes with a mix of parental traits. And that's just what Raphaël Mercier and colleagues have done in a new study on *Arabidopsis thaliana*, a flowering plant in the mustard family with well-known genetics. To stop meiosis, the researchers exploited three differences between sexual and asexual cell division: recombination, which is when replicated chromosomes pair and exchange genetic material; segregation, which is when the chromosome pairs separate; and a second round of cell division.

The researchers created a triple mutant *A. thaliana* that knocks out these differences, resulting in gametes that are clones of the mother plant. Two of the mutants had been isolated previously by other researchers: *Atspo11*-*1*, which prevents pairing and recombination; and *Atrec8*, which modifies chromatid segregation. The researchers had previously shown that the double mutant *Atspo11-1*/*Atrec8* replaces the first meiotic division with a mitotic-like division. However, this double mutant is not fertile.

In this study, Mercier and colleagues identified a gene that prevents the second meiotic division. Calling the gene *osd1* for “omission of second division,” they investigated two *osd1* mutants with different genetic backgrounds. Both mutants (*osd1*-*1* and *osd1*-*2*) appeared normal and were fertile, producing as many seeds per fruit as wild-type plants did. However, crossing the *osd1* mutants with wild-type plants resulted primarily in offspring with three sets of chromosomes (triploid) rather than the usual two (diploid).

This is because all of the male and about 85% of the female gametes had two sets of chromosomes rather than the usual one (haploid).

To learn how *osd1* mutants make diploid gametes, the researchers observed *osd1* chromosomes during meiosis. The first division looked normal, but there were no signs of the second division. This observation was confirmed by a genetic analysis that took advantage of the fact that the two *osd1* mutants are from different strains and so have different genetic backgrounds (No-0 and Ler). The researchers crossed plants that had both *osd1* mutations—meaning they had both types of *osd1* genetic backgrounds—with plants from a third genetic background. The pattern of *osd1* genetic backgrounds in the resulting diploid gametes revealed that *osd1* mutants underwent the first meiotic division but not the second. The gametes contained genetic material from both No-0 and Ler in most parts of their chromosomes, showing that the pairing and recombination associated with the first division had occurred. But only one of the backgrounds (either No-0 or Ler) appeared near the centromeres—a region on the chromosome where sister chromatids come together that undergoes little or no recombination—showing that the sister chromatid separation associated with the second division had not occurred.

Next, Mercier and colleagues combined the *osd1* mutation and the previously known meiosis mutations *Atspo11*-*1* and *Atrec8*. Then, using a similar genetic analysis to that described above, the researchers showed that the resulting triple mutant *osd1*/*Atspo11*-*1*/*Atrec8* produces diploid gametes that are genetically identical to the mother plants. Called *MiMe* for “mitosis instead of meiosis,” this triple mutant has a mitosis-like first cell division and lacks the second cell division. Like *osd1*, but unlike *Atspo11-1*/*Atrec8*, *MiMe* is fertile.

The downside of replacing meiosis with mitosis is that the chromosome number (ploidy) doubles with each generation. As ploidy rises in *A. thaliana*, fertility drops and octoploid *MiMe* mutants produce hardly any seeds. In addition, stopping meiosis is just the first step in achieving plant reproduction from asexual seeds. Apomixis also requires inducing plant embryos and the endosperm that nourishes them to develop without fertilization.

That said, making meiosis like mitosis in *A. thaliana* brings us considerably closer to producing plants that are genetically identical to their mothers. Moreover, the three genes involved are strongly conserved among plants, which means that this part of apomixis is likely to be feasible in other species, including crops.

**d'Erfurth I, Jolivet S, Froger N, Catrice O, Novatchkova M, et al (2009) Turning Meiosis into Mitosis. doi:10.1371/journal.pbio.1000124** [Fig pbio-1000118-g001]

**Figure pbio-1000118-g001:**
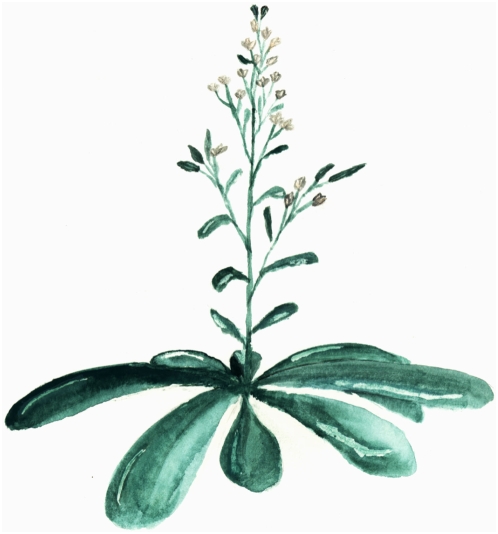
In a first step toward engineering seed clones of important crops, researchers eliminated genetic recombination and chromosome reduction by replacing meiosis with mitosis in *Arabidopsis thaliana.*

